# LEMON: a method to construct the local strains at horizontal gene transfer sites in gut metagenomics

**DOI:** 10.1186/s12859-019-3301-8

**Published:** 2019-12-27

**Authors:** Chen Li, Yiqi Jiang, Shuaicheng Li

**Affiliations:** Department of Computer Science, City University of Hong Kong, Kowloon, Hong Kong SAR, HongKong, China

**Keywords:** HGT, Local strain, Gut metagenomics, Graph

## Abstract

**Background:**

Horizontal Gene Transfer (HGT) refers to the transfer of genetic materials between organisms through mechanisms other than parent-offspring inheritance. HGTs may affect human health through a large number of microorganisms, especially the gut microbiomes which the human body harbors. The transferred segments may lead to complicated local genome structural variations. Details of the local genome structure can elucidate the effects of the HGTs.

**Results:**

In this work, we propose a graph-based method to reconstruct the local strains from the gut metagenomics data at the HGT sites. The method is implemented in a package named LEMON. The simulated results indicate that the method can identify transferred segments accurately on reference sequences of the microbiome. Simulation results illustrate that LEMON could recover local strains with complicated structure variation. Furthermore, the gene fusion points detected in real data near HGT breakpoints validate the accuracy of LEMON. Some strains reconstructed by LEMON have a replication time profile with lower standard error, which demonstrates HGT events recovered by LEMON is reliable.

**Conclusions:**

Through LEMON we could reconstruct the sequence structure of bacteria, which harbors HGT events. This helps us to study gene flow among different microbial species.

## Background

Horizontal Gene Transfer [1, 2] is the movement of genetic materials between organisms other than by the vertical transmission of DNA from parent to offspring [3]. HGTs allow different species to share genomic fragments. Abundant evidence from genomic data now supports that HGT plays an important role in evolution. They are a prevalent and pervasive phenomenon in prokaryotes and are an important source of genomic innovation in bacteria. They are also often observed in unicellular eukaryotes. Recent research suggests that on average 81% of prokaryotes genes have been involved in HGT at some point in their history [4]. Their occurrences in multicellular eukaryotes are rare. However, several significant HGTs are still observed between bacteria and multicellular eukaryotes. Some are even common in specific environments. For example, we have observed HGTs from bacteria to fungi, from bacteria to the coffee borer beetle [5], as well as from virus, bacteria, and fungi to animals [6]. These recent discoveries have reshaped our understanding of evolutionary mechanisms.

HGTs may affect human health through a large number of human microbiota [7], including bacteria, fungi, archaea, and virus. They widely spread on human biofluids and tissues, such as skin, lung, mouth. They are often associated with a range of human diseases and health conditions, from diabetes, colorectal cancer, to autism. The Human Microbiome Project [8] was launched in 2008 to study and understand the human microbiota. Some functions of the human microbiome, including antibiotic resistance and adaption to nutrients [9], are susceptible to HGT events. Mediated by phage, HGT in S.aureus occurs 1000 times more often than was thought, which greatly accelerates S.aureus to evolve resistance to antibiotics [10].

It is necessary to understand HGTs better. However, our current research is mainly focused on inferring ancient (lineage) HGTs from genomic sequences [11]. While the inference result is seriously affected by complex external factors [12]. For example, during the process of evolution, the transferred genome segments had been subjected to loss, mutation and duplication [13]. The inserted genes may also change the expression and functions of the gene around the insertion sites, resulting in very complicated structural variations [14], and temper with the receptor genome’s stabilities [15–17]. These possibilities complicate our detection of the HGTs. Better results can be achieved if we can anticipate these changes and correct for their effects. Recent efforts in human microbiomes provide us with such an opportunity.

LEMON(https://github.com/lichen2018/LEMON) takes use of existing shotgun NGS datasets to detect HGT breakpoints, identify the transferred segments, and reconstruct the local strain, which has complicated structure variation.

## Methods

**Fig. 1:**
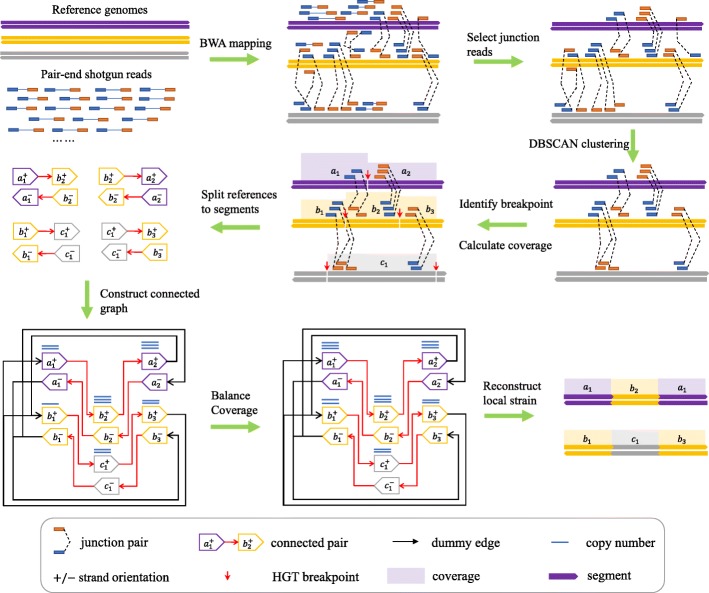
The workflow of reconstructing HGT strains from pair-ends shotgun reads. Firstly, we map paired-end shotgun reads against reference genomes using Burrows Wheeler Aligner (BWA). Then, we select junction reads, whose two sides are mapped to two different references, from the set of mapped reads. Thirdly, by treating junction reads as points on a two-dimensional plane, we apply Density-Based Spatial Clustering of Applications with Noise (DBSCAN) to find candidate HGT breakpoints. Fourthly, we utilize split reads to get the exact positions of HGT breakpoints. Fifthly, according to the detected breakpoints, we could split references into segments, which are linked by junction reads. The coverage of each segment is calculated according to the number of mapped reads on it. Sixthly, we utilize the linked segments to construct a connected graph. By inserting dummy edges, we make the graph fully connected. Seventhly, we balance the coverage of each segment. Finally, we traverse the graph to find local strains. Each local strain should start from the first segment of one receptor and end with the last segment of the same receptor

HGT events result in the integration of DNA segments from one species to another species, which will generate local strains containing DNA segments from different species. Figure 1. illustrates the workflow of LEMON.

### References

Only the assembly results from multiple time-point metagenomics data of one individual can be used to discover the HGT events that exactly occurred between these time points. However, these samples are insufficient in published data, and this method cannot evaluate the difference in HGT events between different samples. To solve this problem, we construct a *reference setS*. We collected all of the bacterial genomes from the National Center for Biotechnology Information (NCBI) [18]. We selected one genome for each taxonomy as a representative genome to reduce the interference from homologous regions based on (1) the genome was annotated as reference or representative by NCBI; (2) or the one has minimal scaffolds number and highest completeness with contamination <10% in The Genome Taxonomy Database (GTDB) taxonomy evaluation results for 109,419 bacterial genomes[19]. The reference set contains 16,093 species with 1,246,881 scaffolds. Given a shotgun genomic read dataset *R*, we utilize BWA[20] to align reads against the references to identify the set of donors and receptors involved. These references with adequately covered segments are then retained. We denote the set of donors and receptors as *D* and *H* respectively.

### Breakpoints and segments

The donor segments and receptor segments interweave in a local strain, separated by HGT *breakpoints*. We need to identify the breakpoints and segments involved in the local strains from *D* and *H*. The data are heterogeneous. According to studies on virus integration [21] and studies on gut metagenomics, breakpoints are surrounded by mutations such as Single Nucleotide Variation (SNV), Copy Number Variation (CNV), short indels and inversions [22]. Hence we detect the breakpoints as follows.

**Fig. 2:**
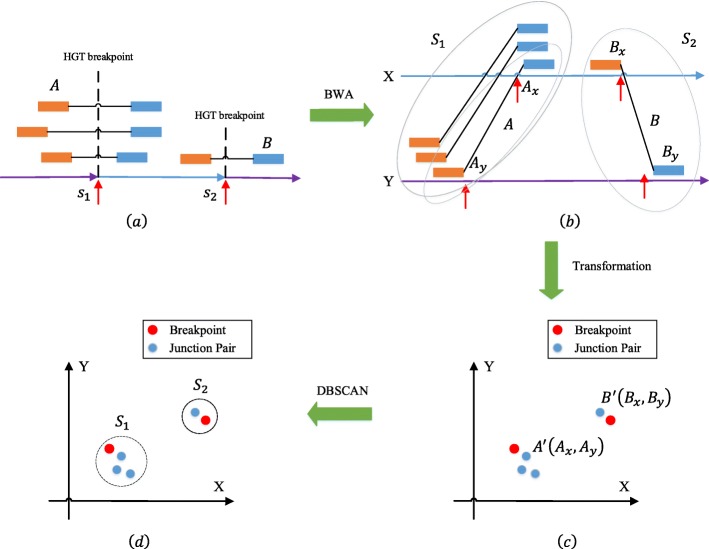
**a** Some paired-end reads cross HGT breakpoints *s*_1_ and *s*_2_. **b** The two sides of one junction pair are mapped to two different references. *B*_*x*_ and *B*_*y*_ are mapped positions of junction pair *B* on reference *X* and *Y* respectively. **c** Furthermore, all junction pairs mapped to the same two references are transformed to points in a coordinate system with their mapped positions as coordinates, e.g. junction pairs A and B are transformed to points *A*^′^(*A*_*x*_,*A*_*y*_) and *B*^′^(*B*_*x*_,*B*_*y*_). **d** We apply DBSCAN using Euclidean distance to cluster the breakpoint pair candidates, such as *S*_1_ and *S*_2_. The maximum diameter of the cluster circle is the insert size of pair-end reads

First, we map paired-end shotgun reads to reference genomes using BWA, here all references are indexed together to generate Burrows–Wheeler Transform (BWT) indexes. If the two sides of a paired-end read are mapped to two different references, we call such a pair a junction pair, such as junction pairs *A* and *B* in Fig. 2a. The mapped positions of each junction pair give us two breakpoint candidates on the respective references. The two mapped positions of each junction pair can be treated as its coordinates on a two-dimensional plane. For example, *B*_*x*_ and *B*_*y*_ in Fig. 2b are mapped positions of junction pair *B* on reference *X* and *Y* respectively. Then pair *B* can be transformed to the point *B*^′^ with coordinates (*B*_*x*_,*B*_*y*_) in Fig. 2c. All junction pairs mapped to the same two references are transformed to points in a coordinate system with their mapped positions as coordinates. We then apply the clustering algorithm DBSCAN [23] using Euclidean distance to cluster the breakpoint pair candidates. A cluster that is supported by at least one junction pair is further subjected to analysis to determine the exact positions of its breakpoints. Next, we identify the split reads which support a cluster. A read is split if it can be partitioned into two parts, with each part mapped to a different reference; we say it *supports* a cluster if the mapped positions belong to the cluster. Each cluster contains multiple breakpoint pair candidates.

To find the exact positions, we use a scoring scheme to rank the candidate positions. The candidate with the highest score is reported as the final breakpoint pair position. The scoring scheme evaluates the split reads that support the cluster. Suppose that there are two references involved in the cluster, *R*_1_ and *R*_2_. Given a candidate genomic positions pair *p*_1_ and *p*_2_ which belongs to *R*_1_ and *R*_2_, respectively, we identify the split reads aligned to *R*_1_ where the alignment terminated at position *p*_1_. Denote the portions of such a split read *s* mapped to *R*_1_ and *R*_2_ as *e*_1_(*s*) and *e*_2_(*s*), respectively. Then, the score is defined according to the alignment qualities of *e*_2_(*s*) against *R*_2_ nearby *p*_2_. The alignment quality *q*_*s*_ of *e*_2_(*s*) is calculated as *m*/*l*, where *m* is the number of matched positions and should be at least 15 bps, *l* is the length of *e*_2_(*s*). The quality score of *p*_2_ is calculated as $1-\sum _{s}\log (1-q_{s})$ of the alignment qualities of the split read which supports the respective cluster. Similarly we calculate the score for *p*_1_. The candidate with the highest score is reported as the final breakpoint pair positions of the cluster.

Each breakpoint involves two segments. Denote the segment pair as $\phantom {\dot {i}\!}\langle u^{x_{u}}, v^{x_{v}}\rangle $, and *x*_*u*_,*x*_*v*_∈{+,−} where + and − respectively indicates the positive and negative strands, and *u*,*v*∈*V*. We call such a pair a *connected pair*. A connected pair is directed; that is, $\phantom {\dot {i}\!}\langle v^{x_{v}}, u^{x_{u}}\rangle \ne \langle u^{x_{u}},v^{x_{v}}\rangle $. Denote the set of connected pairs as *E*, the copy number of *e*∈*E* as *c*(*e*) w.r.t. *R*. It is easy to see that the segments and connected pairs specify a graph *G* as illustrated in Fig. 1.

### Local strains

Assume that there are *k* HGT events captured in *G*. Since each HGT event results in one local strain, there are *k* local strains to be constructed according to *G*. The first and last segments of each local strain are from the same receptor. Without loss of generality let all the first segments and the last segments of the local strains at the integration sites be denoted *B*={*b*_1_,...,*b*_*k*_}, and *T*={*t*_1_,...,*t*_*k*_}, respectively. Denote all the other segments involved in the HGTs as *V*={*v*_1_,...,*v*_*n*_} (excluding *B* and *T*). Next, we estimate the number of copies (copy number) of each segment *v* within *R*, and denote it as *c*(*v*), where *v*∈*B*∪*T*∪*V*. Factors such as species coverage are incorporated into the copy number estimation. For example, if segment *v*_*i*_ is belong to a receptor *r*, the coverage of *v*_*i*_ is *cov*(*v*_*i*_) and the average coverage of *r* is *cov*(*r*), then the initial copy number of *v*_*i*_ is estimated as *c*(*v*_*i*_)=*cov*(*v*_*i*_)/*cov*(*r*).

Our task is to identify the *k* local strains captured in *G*. *B* and *T* can be identified with the input data. We assume that the copy numbers of the first segment and the last segment are the same for each strain, that is, *c*(*b*_*i*_)=*c*(*t*_*i*_), without loss of generality.

### Connectivity

We formulate the problem as a Eulerian circuit problem to find the *k* local strains.

First, we insert dummy edges to transform the solution into a circuit. Without loss of generality, we assume *c*(*b*_*i*_)=*c*(*t*_*i*_),1≤*i*≤*k*. We insert a dummy edge $\langle t_{i}^{+}, b_{i}^{+}\rangle $ with the copy number *c*(*b*_*i*_)−1,1≤*i*≤*k*. The edges $\langle t_{i}^{+}, b_{i+1}^{+}\rangle, 1\le i\le k-1$, and edge $\langle t_{k}^{+}, b_{1}^{+}\rangle $ are inserted with copy number 1.

Second, we insert edges and nodes to ensure connectivity. In the ideal case, for each vertex *v*∈*V*, there should be a path that starts from a source node in *S*, passes through *v*, and ends up at some target in *T*. However, due to sequencing errors, edges or nodes can be missing or spuriously introduced. If no target and source can reach a node *v*, we remove *v* and its adjacent edges from *G*. If a path exists from some vertex *s* to *v*, but no path exists from *v* to a target *t*, we insert vertices and edges to form a path from *v* to *t*. The inserted edge candidates are taken from the reference sets *D* and *S*. If *v* belongs to the same reference as *t*, this would suffice to reconstruct the path. Otherwise, we add edges that connect *v* and some nodes on the reference of *t*. In both situations, we use the minimum number of edges required. All the introduced edges and vertex are assigned a copy number of 1.

### Balancing the graph

Denote the set of inbound edges of *u* as *in*(*u*) and the outbound edges of *u* as *out*(*u*). That is, *in*(*u*)={*u*|(*v*^*x*^,*u*^+^),(*u*^−^,*u*^*x*^)∈*E*,*x*∈{+,−}},*out*(*u*)={*u*|(*v*^*x*^,*u*^−^),(*u*^+^,*v*^*x*^)∈*E*,*x*∈{+,−}}. The *in-copy* and and *out-copy* of a vertex *v* are defined as $c_{in}(u)=\sum _{e\in in(u)} c(e)$ and $c_{out}(u)=\sum _{e\in out(u)} c(e)$. In the ideal case, we should have *c*_*in*_(*u*)=*c*(*u*)=*c*_*out*_(*u*), but this may be broken due to experimental and sequencing errors.

We propose an integer linear programming (ILP) approach to optimize the degree balance property. First, assign each segment *u* (respectively *μ*) to a target copy number *t*(*u*) (respectively *t*(*μ*)) according to Eq. 1c (respectively 1d), to satisfy the degree balance property (Eq. 1b). Then, the following program minimizes the disagreement between the assignment copy number and the target copy number (Eq. 1a).

1a $$\begin{array}{*{20}l} \text{minimize} \quad & \sum_{u}\epsilon_{u}+\sum_{\mu}\epsilon_{\mu} &  \end{array} $$

$$\begin{array}{*{20}l} \text{subject to} \quad & c_{in}(u)=c(u), c_{out}(u)=c(u), \end{array} $$

1b $$\begin{array}{*{20}l} &\forall u\in S\cup V\cup T  \end{array} $$

1c $$\begin{array}{*{20}l} & -\epsilon_{u} \le c(u)-t(u)\le\epsilon_{u},\\ &\forall u\in S\cup V\cup T  \end{array} $$

1d $$\begin{array}{*{20}l} & -\epsilon_{\mu} \le c(\mu)-t(\mu)\le\epsilon_{\mu}, &\forall \mu\in\! J  \end{array} $$

1e $$\begin{array}{*{20}l} & \epsilon_{v}, \epsilon_{\mu}\in R^{+} &  \end{array} $$

1f $$\begin{array}{*{20}l} & t(u), t(\mu)\in\mathcal{I}, t(u)\ge 1, t(\mu)\ge 1 &  \end{array} $$

### Finding Eulerian circuit

It can be shown that a Eulerian circuit to the graph constructed as illustrated in Fig. 1 gives a solution to our local strain reconstruction problem. However, the problem may yield multiple solutions. Each local strain should start from the first segment of one receptor and end with the last segment of the same receptor as illustrated in Fig. 1. Each reconstructed strain may contain several HGT events. Let the number of HGT events that contributes to the local strains *i* be denoted as *h*_*i*_. We choose a solution in which each reconstructed strain *i* has *h*_*i*_ as large as possible.

### Evaluation metrics

To evaluate the performance of LEMON, we construct true local strains containing transferred segments and use LEMON to recover them. We propose two metrics Reconstruction Accuracy and Detection Rate to measure results.

We denote the true local strains as $\left \{ H_{i}\right \}_{i=1}^{n}$, where *n* is the number of receptors, and each true local strain {*H*_*i*_} consists of *m*_*i*_ segments, that is, $H_{i}=\left \{ s_{j}^{H_{i}}\right \}_{j=1}^{m_{i}}$. We take the simulated reads as input of LEMON and construct reconstructed local strains $\left \{ H_{i}\right \}_{i=1}^{n}$, where *h*_*i*_ is the reconstructed local strain which has the same receptor of *H*_*i*_; if *H*_*i*_ doesn’t have *h*_*i*_ in the result, we set *h*_*i*_=*∅*. The segments in *h*_*i*_ are denoted $s_{j}^{h_{i}}$, that is, $h_{i}= \left \{ s_{j}^{h_{i}}\right \}_{j=1}^{l_{i}}$. We apply Smith-Waterman algorithm to measure the similarity between segment sequences of *H*_*i*_ and *h*_*i*_. $\forall s_{j}^{h_{i}}\in h_{i}$ and $\forall s_{j}^{H_{i}} \in H_{i}$, we consider $s_{j}^{h_{i}}$ and $s_{j}^{h_{i}}$ to be matched if and only if the breakpoint pair positions of $s_{j}^{h_{i}}$ are both located within 20 bp around the breakpoint pair positions of $s_{j}^{h_{i}}$. In our experiments, the parameters *match*_*score* and *mismatch*_*score* of Smith-Waterman algorithm are set as 1 and -1, respectively.

The reconstruction accuracy *RA*_*i*_ of *h*_*i*_ is defined in formula (2),

2 $$ RA_{i}= \left\{\begin{array}{cc} \frac{SW(h_{i},H_{i})}{m_{i}*match\_score}, & \text{\(h_{i} \neq \emptyset\)}\\ 0, & \text{\(h_{i}=\emptyset\)}. \\ \end{array}\right.   $$

Here, *SW*(*h*_*i*_,*H*_*i*_) is the alignment score of *h*_*i*_ and *H*_*i*_, while *m*_*i*_ is the number of segments in *H*_*i*_. When all segments are matched in *h*_*i*_ and *H*_*i*_, which means all transferred segments are recovered. So *SW*(*h*_*i*_,*H*_*i*_) is equal to *m*_*i*_∗*match*_*score*, that is *RA*_*i*_=1.

We set the number of repetitions *N* as 8 and define the mean value of reconstruction accuracy $\bar {RA}$ as follows,

3 $$ \bar{RA} = \frac{1}{N}\sum_{k=1}^{N} \frac{\sum_{i=1}^{N}RA_{ik}}{n_{k}}  $$

*n*_*k*_ denotes the number of non-empty *h*_*i*_ in the *j*-th repetitions.

An acceptable detection of one transferred segment should have its breakpoint pair located within 20 bp [24] of the true breakpoint pair as mentioned in “Breakpoints and segments” section. The *Detection Rate* is defined as the rate of the number of acceptable recovered transferred segments and the number of all transferred segments. The average detection rate is the average of 8 repetitions for each test.

### Software parameter setting

Most third-party tools used in this article are set default parameters, including BWA, LUMPY [25], iRep [26], and STAR-Fusion [27]. The parameters of DBSCAN are *eps*, which is the maximum radius of one cluster, and *min*_*sample*_, which is the minimum number of points in one cluster. In our paper, *eps* is set as the average insert size. *min*_*sample*_ is set as 1.

## Results

### HGT events detection in simulated human gut microbiomes

To simulate human gut microbiome with different complexity as mentioned in “Local strains” section, we constructed 5 simulated microbiomes containing 160, 320, 640, 1280, 2560 species, respectively. For each simulated microbiome, 5 different amounts (20, 40, 60, 80, 100) of HGT events were generated. For each HGT event, we randomly selected two reference sequences as the receptor and donor respectively. On the donor, we randomly selected one 10k bp sequence region as a transferred segment and inserted it to a randomly selected insertion position on the receptor. In this simulation, each HGT event contained one transferred segment. We denoted the new receptor sequence containing transferred segments as the true local strain. All true local strains were used to generated 20X paired-end reads with WGSIM [28] as an input of LEMON. Eight repetitions were performed for every test.

In order to prove the performance of LEMON, we compared its performance with another popular breakpoint detection-based structural variant discovery software LUMPY.

**Fig. 3:**
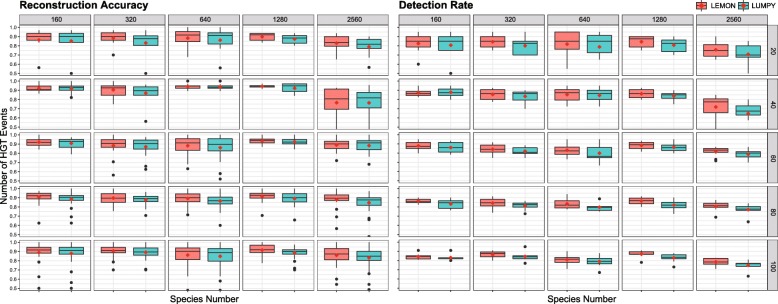
Comparison of Reconstruction Accuracy and Detection Rate between LEMON and LUMPY under different simulated conditions. The red dot in each boxplot is the mean value. Most mean values of Reconstruction accuracy and Detection rate achieved by LEMON are higher than those achieved by LUMPY

Figure 3 illustrates a Comparison of Reconstruction Accuracy and Detection Rate between LEMON and LUMPY under different simulated conditions. The red dot in each boxplot denotes the mean value. As we can see, most mean values of Reconstruction Accuracy and Detection Rate achieved by LEMON are higher than those achieved by LUMPY, which demonstrates that LEMON can reconstruct more accurate strains and detect more transferred segments than LUMPY.

### HGT breakpoints detection

**Fig. 4:**
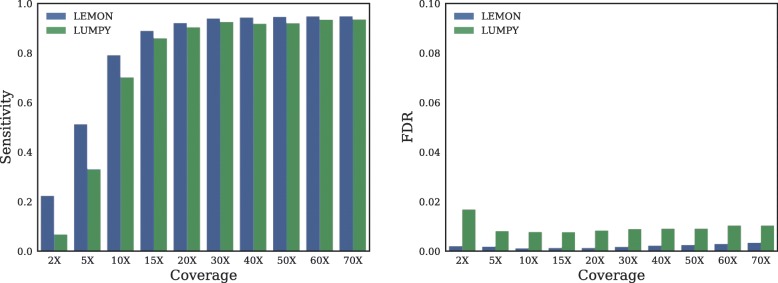
Sensitivity and FDR comparison between ours and LUMPY. At low coverage level, LEMON is more sensitive than LUMPY. At higher coverage level, LEMON remains slightly better than LUMPY. LEMON has lower FDR than LUMPY across different coverage levels

In order to evaluate the performance of LEMON in HGT breakpoints detection as mentioned in “Breakpoints and segments” section, we applied it to local strains with different coverage and compared the performance with LUMPY. HGT events with random receptors, donors and breakpoints were generated in 100 randomly selected microbials, resulting in 60 local strains with 4260 HGT breakpoints. The HGT breakpoint is the insertion position of donor segments such as *s*_1_ and *s*_2_ in Fig. 2a. The paired-end reads generated from these local strains with 10 different values (2X, 5X, 10X, 15X, 20X, 30X, 40X, 50X, 60X, and 70X) of depth were input of LEMON and LUMPY. The performance is measured in terms of Sensitivity and False Discovery Rate (FDR) in breakpoints detection, and the bearing bias is 20 bp. If the distance between the detected breakpoint position and the true position is larger than 20 bp, we treat this detected position as one false detected position. Then if the distance is less than 20 bp, the detected position is treated as one true detected position. Therefore, FDR is the rate of false discovered positions among all discovered breakpoint positions. Sensitivity is the rate of true detected positions among all true breakpoint positions. LEMON has higher sensitivity and lower FDR than LUMPY across different coverage levels as illustrated in Fig. 4. At low coverage level, e.g. 2X and 5X coverage, LEMON can detect 22.39% and 51.19% of all HGT breakpoints, whereas LUMPY can detect 6.86% and 31.12% of all HGT breakpoints. At a higher coverage level, LEMON remains slightly better than LUMPY. For example, from 10X to 70X coverage, the detection sensitivity of LEMON ranges from 79.03 to 94.79%, whereas the detection sensitivity of LUMPY ranges from 70.06 to 93.47%. LEMON has lower FDR than LUMPY across different coverage levels, for example, at 2X, the FDR of LEMON is 0.002, while the FDR of LUMPY is 0.016. At 70X, the FDR of LEMON and LUMPY are 0.0034 and 0.010 respectively. Therefore, LEMON can detect more accurate breakpoints than LUMPY.

### HGT strains reconstruction with complicated HGT event structure

In this simulation, we set the number of species s to 2560 and the number of HGT events to 100. In order to simulate a complicated HGT event structure, we changed the number of transferred segments in each HGT event from 1 to 5. Simulated paired-end reads were generated from true local strains by using WGSIM at 5X, 10X, 20X, and 30X coverage. 4 repetitions were performed.

**Fig. 5:**
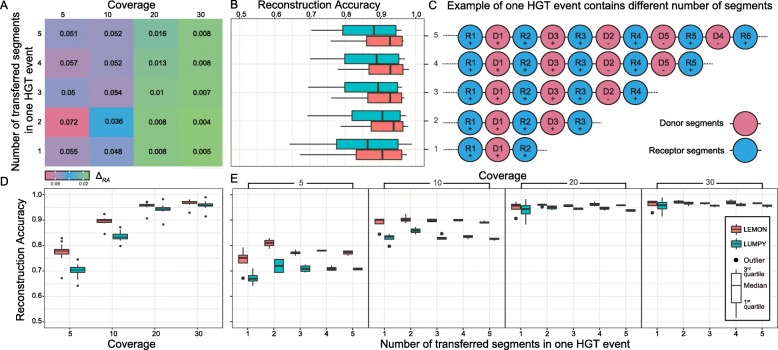
Comparison of Reconstruction Accuracy between LEMON and LUMPY under different levels of coverage and number of transferred segments in one HGT event. **a** All *Δ*_*RA*_ are positive and *Δ*_*RA*_ decreases as coverage increases. **b** demonstrates that under different complexity of the HGT event, *RA*_*LEMON*_ is better than *RA*_*LUMPY*_. **c** gives an example of one HGT event containing the different number of transferred segments. **d** compares Reconstruction Accuracy across different levels of coverage. **e** demonstrates that at different coverage level, LEMON has higher RA than LUMPY under different complexity of HGT event

Figure 5 shows the Comparison of Reconstruction Accuracy between LEMON and LUMPY under different coverage and number of transferred segments in one HGT event. We use *Δ*_*RA*_=*RA*_*LEMON*_−*RA*_*LUMPY*_ to measure the performance difference between LEMON and LUMPY. If *Δ*_*RA*_ is positive, LEMON achieves a higher *RA* than LUMPY. As we can see from Fig. 5a, all *Δ*_*RA*_ are positive and *Δ*_*RA*_ decreases as coverage increases. Figure 5b demonstrates that under different complexity of HGT event, *RA*_*LEMON*_ is better than *RA*_*LUMPY*_. Figure 5c gives an example of one HGT event containing a different number of transferred segments. Figure 5d and e demonstrate that LEMON has better performance than LUMPY across different levels of coverage, especially at low coverage levels, such as 5X and 10X.

**Fig. 6:**
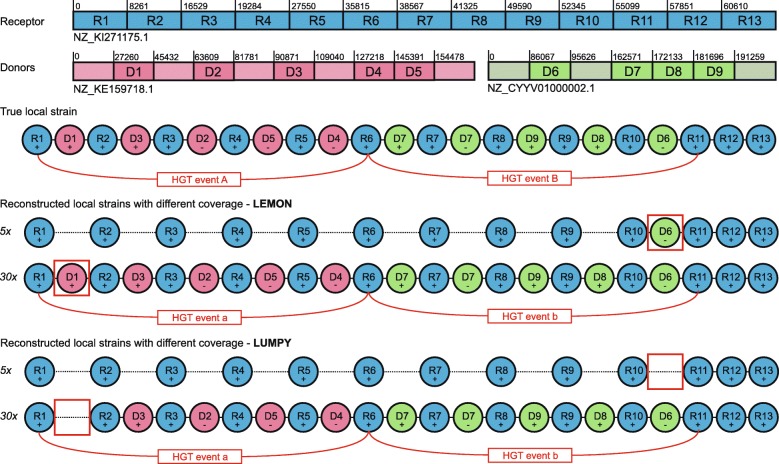
Comparison of local strains reconstructed by LEMON and LUMPY at 5X and 30X coverage levels. The true local strain contains two HGT events A and B. Each HGT event has five transferred segments. +/- denotes the orientation of the segment. At 5X, LEMON detects one transferred segment Green -D6, while LUMPY fails to detect anyone transferred segment. At 30X, LEMON detects all transferred segment and has reconstructed the same structure as the true local strain, while LUMPY fails to detect Red +D1

In Fig. 6, we compare local strains reconstructed by LEMON and LUMPY at 5X and 10X coverage levels. The true local strain contains two HGT events A and B. Each HGT event has five transferred segments. +/- in each segment represents the forward/reverse direction of the segment. At 5X, LEMON detects one transferred segment Green -D6 and the RA is 0.6087, while LUMPY fails to detect anyone transferred segment and the RA is 0.5238. At 30X, LEMON detects all transferred segment and the RA is 1.0, which means LEMON has reconstructed the same structure as the true local strain, while LUMPY fails to detect Red +D1 and the RA is 0.9565. Therefore, LEMON could detect more transferred segments than LUMPY and reconstruct more accurate strains across different coverage levels.

### Highly complex HGT structures do exist in real metagenomic data

**Fig. 7:**
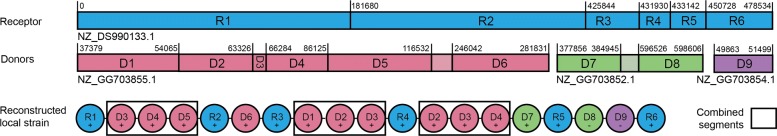
Reconstructed local strain of NZ_DS990133.1 in F-5. NZ_DS990133.1 is the receptor. NZ_GG703855.1, NZ_GG703852.1, and NZ_GG703854.1 are donors. Numbers above the receptor denote insertion positions. Numbers above donor denote the start/end positions of segments. +/- denotes the orientation

We applied LEMON on a recently released metagenomic dataset [29] to reconstruct local strains containing HGT events. Some reconstructed local strains have complex HGT events, such as the reconstructed local strain of NZ_DS990133.1 in sample F-5 as shown in Fig. 7).

**Fig. 8:**
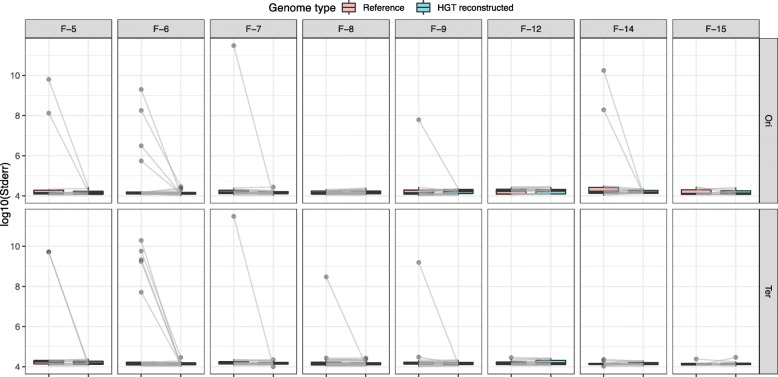
The change of Standard Error at the origin and terminus of replication before and after HGT reconstructions. Standard error has scaled with log10. Boxplots are scaled standard error distribution at origin and terminus. Red boxes represent results calculated based on the original reference of receptor containing no HGT event and blue boxes represent results calculated based on the reconstructed reference of receptor containing HGT events. Grey lines denote some reconstructed local strains have much lower Standard Error

As we can see from Fig. 8, segments from one donor are not always inserted into the receptor as a whole. Sometimes they are inserted into the receptor together with segments from other donors. For example. Segments D1-D2-D3 and D2-D3-D4 from NZ_GG703855.1 are inserted at the position of 181,680 bp and 431,930 bp on NZ_DS990133.1 respectively. The D2-D3-D4 from NZ_GG703855.1 together with D7 from NZ_GG703852.1 is inserted at the position of 433,142bp on the receptor. And the D8 from NZ_GG703852.1 together with D9 from NZ_GG703854.1 is reverse inserted at the position of 450,728bp on the receptor, which demonstrates the complexity of HGT events.

### Local strains reconstructed by LEMON can assist replication timing profile restoring

We used iRep to estimate the replication timing profile of each bacterium in metagenomics data [29]. iRep utilizes linear regression to evaluate the coverage distribution across the genome to determine the PTR (peak-to-trough ratio), which is the ratio between the coverage at the origin and terminus of replication. However, due to the limitations of the reference sequence and the low sequencing depth of most species, we typically got very few replication timing profiles in a single metagenomics sample.

We applied iRep to evaluate two coverage distributions for each receptor. The first coverage distribution is evaluated based on the original reference of the receptor containing no HGT event. The second coverage distribution is evaluated based on the reconstructed reference of the receptor containing HGT events. According to the two coverage distributions, we estimated two replication timing profiles(including PTR value, predicted origin, and terminus position) for each receptor. Since iRep utilizes the regression method to estimate replication timing profiles, we use Standard Error to measure the accuracy of the estimated replication timing profiles. Figure 8 demonstrates the change of Standard Error at the origin and terminus of replication before and after reconstructions, some reconstructed local strains have much lower Standard Error, which means that LEMON help to reconstruct strains containing HGT events with more accurate restoring replication timing profile.

### Verifying HGT breakpoints with gene fusion breakpoints detected from metatranscriptome data

In order to verify the HGT breakpoints detected by LEMON, we analyzed the IBD (Inflammatory Bowel Disease) data set published by HMP (Human Microbiome Project) [30]. In addition to metagenomic sequencing data, some samples in this data set have corresponding metatranscriptome sequencing data. The HGT breakpoints on DNA should cause some gene fusions in RNA. We used STAR-Fusion, the current state-of-the-art tool in gene fusion detection, on the metatranscriptome data to obtain gene fusion results. These results were compared with the breakpoint results in the corresponding metagenomic data obtained by LEMON and LUMPY. Three HGT breakpoints that have close gene fusions results within 200 bp were found among 17 pairs of metagenomics and metatranscriptome data as illustrated in Table 1. HGT breakpoints detected by LEMON and LUMPY have different shift distances away from fusion points detected by STAR-Fusion. This may validate that some gene fusions in bacterial chromosomes are caused by HGT.

**Table 1:** Statistic table of HGT breakpoints type in strain results of eight samples

Sample	Total	Non-coding	Candidate gene	Candidate gene
	breakpoints	breakpoints	fusion points	fusion points ratio
F-5	1024	227	228	22.3%
F-6	390	248	18	4.6%
F-7	437	261	24	5.5%
F-8	575	322	28	4.9%
F-9	230	157	11	4.8%
F-12	459	326	11	2.4%
F-14	364	243	14	3.8%
F-15	377	230	19	5.0%

The reads supporting the breakpoint, NZ_DS981501.1:4185-NZ_CP015401.2:963348, are shown in Additional file 1. The shift distance between the HGT breakpoint and the breakpoint obtained by STAR-Fusion is 10 bps. The shift sequence regions on the two reference sequences, such as TAATGGTTAG and TAATGGTTCA in Additional file 1, are almost the same.

We identify 3 main reasons for discrepancies between STAR-Fusion-detected gene fusion breakpoints and our HGT breakpoints:

1) The results of STAR-fusion are based on STAR aligner, while our algorithm is based on BWA. STAR aligner and BWA employ different alignment algorithm, giving rise to different breakpoints results;

2) Limited sequencing data. The amount of metagennomics sequencing data in the IBD data set is around 5G per sample, and the amount of metatranscriptome data is 2G per sample. This is insufficient for the statistical significance required for finding all the breakpoints;

3) Based on our statistics in Table 2, most of HGT breakpoints occur in the non-coding region.

**Table 2:** Detail on the breakpoints of three gene fusion results which have close HGT breakpoints (HGT breakpoints located in 200 bp upstream or downstream around gene fusion points)

Sample		H4009C2	C3003C3	C3003C3
Upstream		NZ_GG703852.1	NZ_ACEP01000119.1	NZ_DS981501.1
Downstream		NZ_JRNC01000070.1	NZ_ACEP01000074.1	NZ_CP015401.2
Upstream breakpoint	HGT	751372	25274	4185
	LUMPY	751114	25275	4220
	STAR-Fusion	751131	25282	4195
Downstream breakpoint	HGT	199	27544	963348
	LUMPY	127	27558	963349
	STAR-Fusion	223	27588	963357
Upstream gene name		NZ_GG703852.1_gene2906	NZ_ACEP01000074.1_gene1544	NZ_DS981501.1_gene822
Downstream gene name		NZ_JRNC01000070.1_gene2118	NZ_ACEP01000119.1_gene553	NZ_CP015401.2_gene767

In summary, it is reasonable to find only 3 matching breakpoints in 17 pairs of data.

## Conclusions and discussion

In this paper we present LEMON, a novel HGT discovery software that can detect HGT events and reconstruct strains containing multiple HGT events with complicated structural variation.

Using LEMON to reconstruct the sequence structure of bacteria allows us to study the metagenomics problem from the sequence level, thus no longer subjected to the comparison of abundance. For example, since HGT is the fundamental mechanism for the spread of antibiotic resistance in bacteria, by utilizing LEMON we could detect transferred Antibiotic Resistance Genes (ARG)[31], determine the corresponding donors and receptors, and reconstruct strains of receptors, which harbor the transferred ARG. Therefore, we could get a better understanding of the transfer mechanism of ARG among bacteria.

However, as the amount of sequencing data is generally insufficient for current metagenomics analysis, challenges remain in identifying the HGTs sensitively and accurately. This results in several shortcomings in LEMON. First, LEMON remains weak in finding HGT between the sequences that do not exist in the reference genome. Second, because we only consider the reads of unique mapping, the HGT on the repeat region cannot be identified.

At present, our reference set only contains the genome of bacteria. However, human gut microbiome also contains other microorganisms such as fungi and viruses. Therefore, a reference library that contains sequences of bacterial, viral and fungal more completely would be highly desirable for HGT analysis of the microbiome.

## Additional material

Additional file 1 Detailed reads mapping result at HGT breakpoints NZ_DS981501.1:4185 - NZ_CP015401.2:963348. Top-left is upstream genome; top-right is downstream genome. STAR-Fusion determined genes around gene fusion points are annotated with border bar. Red lines represent breakpoints detected from metagenomics data with HGT algorithm. Blue lines represent breakpoints detected from metatranscriptome data with STAR-Fusion. In red rectangle, top sequence with base name is local strain constructed with HGT breakpoints information, and other color bars are metagenomics reads support breakpoints. In blue rectangle, top sequence with base name is local strain constructed with gene fusion breakpoints, other color bars are metatranscriptome reads support those breakpoints.

## Data Availability

The real metagenomics dataset for local strains recontruction was deposited to Sequence Read Archive (Bioproject: PRJNA393237). IBD dataset was published by HMP and available on the Sequence Read Archive (BioProject: PRJNA389280)

## Abbreviations

ARG: Antibiotic resistance genes
BWA: Burrows wheeler aligner
BWT: Burrows–wheeler transform
CNV: Copy number variation
DBSCAN: Density-based spatial clustering of applications with noise
FDR: False discovery rate
GTDB: Genome taxonomy database
HGT: Horizontal gene transfer
HMP: Human microbiome project
IBD: Inflammatory bowel disease
ILP: Integer linear programming
iRep: Index of replication
NCBI: National center for biotechnology information
NGS: Next-generation sequencing
PTR: peak and trough of replication
SNV: Single nucleotide variation
WGSIM: Whole-genome sequencing read simulator
