# Supplementary figures and images for: LEMON: a method to construct the local strains at horizontal gene transfer sites in gut metagenomics
Source: BMC Bioinformatics. 2019 Dec 27;20(Suppl 23):702. doi: 10.1186/s12859-019-3301-8 (PMC6933643; doi:10.1186/s12859-019-3301-8)

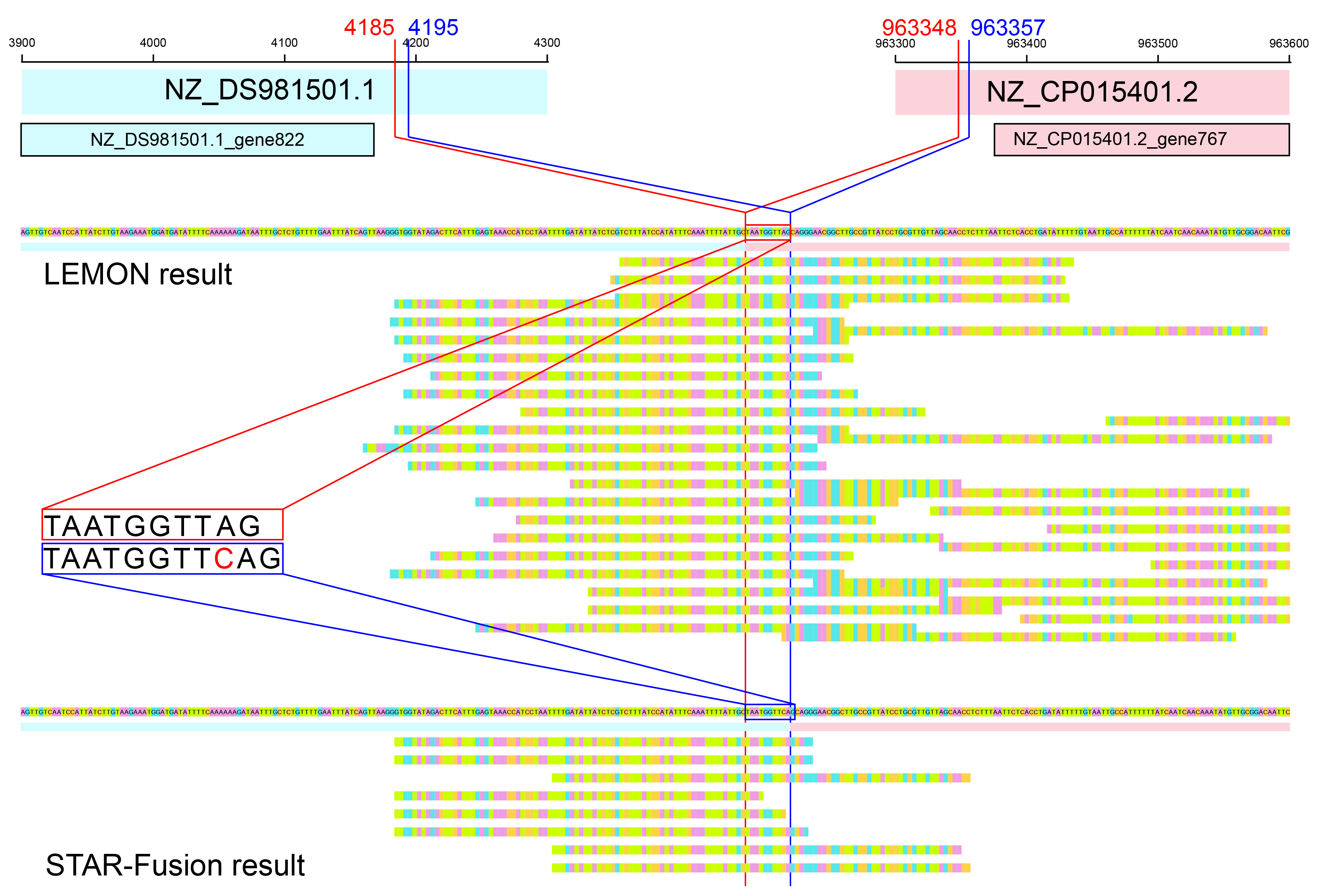

Supplement: Supplementary file 1 — Detailed reads mapping result at HGT breakpoints NZ_DS981501.1:4185 - NZ_CP015401.2:963348. Top-left is upstream genome; top-right is downstream genome. STAR-Fusion determined genes around gene fusion points are annotated with border bar. Red lines represent breakpoints detected from metagenomics data with HGT algorithm. Blue lines represent breakpoints detected from metatranscriptome data with STAR-Fusion. In red rectangle, top sequence with base name is local strain constructed with HGT breakpoints information, and other color bars are metagenomics reads support breakpoints. In blue rectangle, top sequence with base name is local strain constructed with gene fusion breakpoints, other color bars are metatranscriptome reads support those breakpoints. [file 12859_2019_3301_MOESM1_ESM.png]
